# Facilitators and barriers for the implementation of telemedicine from a local government point of view - a cross-sectional survey in Germany

**DOI:** 10.1186/s12913-021-06929-9

**Published:** 2021-09-06

**Authors:** Maja Maria Weißenfeld, Katja Goetz, Jost Steinhäuser

**Affiliations:** Institute of Family Medicine, University Hospital Schleswig-Holstein, Campus Luebeck, Ratzeburger Allee 160, 23538 Luebeck, Germany

**Keywords:** Telemedicine, Local politics, General practitioner shortage, Primary care

## Abstract

**Background:**

Telemedicine offers additional ways of delivering medical care, e.g., in primary care in rural areas. During the last decades, projects including telemedicine are being implemented worldwide. However, implementation of telemedicine is in some countries, e.g., Germany somewhat slower compared to northern European countries. One important part of successful implementation is to include the citizen perspective. The aims of this study were to explore the perception of representatives of the local government regarding telemedicine in the context of a perceived GP shortage and to tailor future telemedicine offers according to these perceived needs.

**Methods:**

Considering the multidisciplinary assessment suggested by the Model for Assessment of Telemedicine a questionnaire with 19 questions was developed by identifying determinants of telemedicine out the literature. After pre-testing, the questionnaire was sent to all 2199 mayors from the federal states of Schleswig-Holstein (North Germany) and Baden-Württemberg (South Germany) as representatives of the citizens (cross- sectional study; full population survey). The final questionnaire contained sections for socio-demographic data, telemedicine and perceived GP shortage. All responses from November 2018 until 2019 were included and analyzed descriptively.

**Results:**

The response rate was 32% (*N* = 699), of which 605 were included in the analysis. A majority of the participants stated they live in a rural area and 46% were in the office for up to 8 years. The mayors had predominantly a positive perception about telemedicine (60%) and 76% of them stated, their community would benefit from telemedicine. A GP shortage was reported by 39% of the participants. The highest risk of telemedicine was seen in misdiagnosing. In case of an emergency situation 291 (45%) of the participants considered data privacy as not as relevant. Mayors from a community with a perceived GP shortage had a more negative perception regarding telemedicine.

**Conclusion:**

The acceptance of telemedicine is rapidly rising compared to former studies. Communities with a perceived GP shortage had a more negative perception. Barriers like data security concerns were seen as less important in case of an emergency. The highest risk of telemedicine was seen in misdiagnosing. These findings need to be considered in designing future telemedicine offers.

**Supplementary Information:**

The online version contains supplementary material available at 10.1186/s12913-021-06929-9.

## Background

Telemedicine is a part of delivering health care using e-health. E-health describes the general use of electronic devices and digital data in health care. The term telemedicine subsumes care concepts in which medical services are provided over spatial distances or different time zones, using information and communication technologies [1]. Telemedicine offers an opportunity to improve access to care, especially in remote areas and therefore has the potential to optimize health care [2]. Furthermore, when it comes to the topic of access to medical professionals, digitalization seems to offer new ways of care, also- and especially for rural areas [3, 4].

Telemedicine is used throughout various medical specialties. The benefits of telemedical services are the accessibility and the reduction of medical errors [5]. However, the clinical use of telemedicine varies between different countries. In Germany in February 2020 about 1.700 practices offered telemedicine. In April 2020 this number grew up to 25.000 practices due to the SARS-CoV-2 pandemic [6]. Until the pandemic telemedicine was not as implemented as a standard as in other countries in Europe like Estonia [7]. Especially regarding the use of electronical prescriptions and electronical patient record Germany is behind.

In 2017, 76% of the hospitals in the United States of America connected with patients via telemedicine [8]. There are several known determinants influencing the implementation and acceptance of telemedicine. The existence of a broadband internet connection is crucial. According to the German Federal Ministry of Transport and Digital Infrastructure, some rural areas still don’t have access to a private broadband internet connection [9]. Furthermore, important determinants are qualified and willing medical staff and financing of health care that is delivered this way [10, 11].

In Germany, the National Association of Statutory Health Insurance (SHI) Physicians is responsible for the medical supply of the population [12]. Among others, they distribute districts where physicians can settle, in order to regulate well-adjusted medical access [13].

Local government has a so-called “supply-duty” it can negotiate with the Association of SHI Physicians, therefore their perception about telemedicine is relevant. In times of a general physician shortage, the demand for general practitioners (GPs), especially in rural areas is growing [14]. Additionally, there is a growing demand on medical professionals due to several reasons such as the demographic change [15]. In a society of a longer lifespan, the growing portion of multimorbid patients need to be treated by also older doctors [14].

In this situation, mayors of rural communities do start to get involved to secure the local health professional demand of their community [14, 16, 17]. However, they seemed rather skeptical about telemedicine use in former studies. Especially the perceived disadvantages for older people, impersonal treatment and poor availability of necessary technology were issues of concern [2, 18].

The Model for Assessment of Telemedicine (MAST) specifies that an assessment of telemedicine should include effects or outcomes within seven different domains including safety, clinical effects, patient perception, economic aspects, organizational aspects and ethics and legal aspects [19].

By that the MAST offers a framework of relevant aspects by implementing telemedicine care.

Ione MAST domain suggests a multidisciplinary assessment including patients. Therefor mayors seem to by a crucial target group as they are patients themselves and influence the propaganda regarding telemedicine at a local level.

Aim of this study was to explore the spectrum of perception of representatives of the local government regarding telemedicine and the perceived general practitioner shortage in two different federal states to tailor future telemedicine offers according to the perceived needs.

## Methods

### Design and participants

The study was confirmed to the STROBE-Guidelines (Strengthening the Reporting of Observational Studies in Epidemiology) [20]. This cross-sectional study was conducted in Schleswig-Holstein, a federal state in northern Germany and Baden-Württemberg, a federal state in southern Germany. This was a full survey. Inclusion criteria were, that participants had to be elected mayors in one of the two federal states. For the recruitment of mayors, the two municipal head organizations of the “Schleswig-Holsteinischer Gemeindetag” and the “Gemeindetag Baden-Württemberg” were informed. The addresses of the mayors were openly available by the information of the official websites of each community. All 2199 mayors from these two federal states were invited to join the survey from November 2018 until February 2019. The return of the anonymous paper-based questionnaire was classified as informed consent.

### Measure

For the evaluation of the spectrum of perception of representatives of the local government regarding telemedicine it was necessary to develop a questionnaire. Therefore, a pragmatic literature search in the Medline database PubMed with the terms “telemedicine AND barriers AND facilitators” and “Telemedizin” was performed in April 2018. Included were all articles in German or English language and published within the last 5 years (2012 to 2017), which either implied the current status of telemedicine or the determinants of the implementation of telemedicine. Due to the literature research performed by MW six papers, published from 2012 to 2017 were included [3, 11, 12, 21–23]. Out of these the determinants were extracted, discussed and clustered by MW and JS using the domains of the Model for Assessment of Telemedicine (MAST) [19] and transferred into a first version of the questionnaire.

This version was piloted with two representatives of the two municipal head organizations in both federal states, the “Schleswig-Holsteinischer Gemeindetag” and the “Gemeindetag Baden-Württemberg”. Feedback was collected using the think-aloud technique [24]. Main questions were understandability and clearness of the items. During this pilot process, the participant also enters their comments on the questionnaire while completing it. This method helps review the understandability of the questions and either change or delete certain questions.

The final questionnaire consisted of 19 questions in three sections: telemedicine, GP shortage and socio-demographics. Socio-demographics included length of term of office, gender, number of inhabitants. The size of the community was measured by the number of inhabitants. The Federal Office of Construction and Urban Research (BBSR) defined a community of more than 5000 inhabitants as a city and with less as municipality/rural area [25]. The items could either be answered with a 5-point Likert-Scale (1 = strongly agree and 5 = strongly disagree), with yes/no- options or with a free text.

As an alternative for non-participation, a short-response-sheet was offered which consisted of five items (short version). These were: gender, age, rural location, length of term of office and reasons for the non-participation.

Both questionnaires (in German language) can be requested from the authors of this manuscript.

#### Statistical analysis

Statistical analyses were performed using SPSS 25.0 (Inc., IBM). Continuous data was summarized using means and standard deviations. Categorical data was presented as frequency counts and percentage. Content validity was assessed by the exploratory factor analysis. The principal component analysis with extraction of component loadings was performed to test the dimensionality of the individual sections. The component loadings were subjected to Varimax rotation, and their number was determined by eigenvalues > 1. Furthermore, sample suitability was evaluated with the Kaiser-Meyer-Olkin (KMO) criterion, and Bartlett’s test was performed to examine sphericity (*p* < 0.05) [26]. To measure the reliability internal consistency for each scale was assessed using Cronbach’s alpha, which indicates whether an item of a scale is appropriate for assessing the underlying concept of the scale [27]. Values for Cronbach’s alpha range from 0 to 1. The closer they are to 0, the less related the items are to one another. Values > 0.8 represent good internal consistency, while values > 0.6 and values > 0.4 represent acceptable consistency and poor internal consistency, respectively. Differences between men or women, mayors from rural areas or cities, mayors from communities with a perceived doctor’s shortage, and mayors with more years of service than with less years of services concerning perception towards telemedicine were analyzed using non-parametric Mann-Whitney-U test. The incidence of missing data < 10% was negligible for the data analysis. An alpha level of *P* < 0.05 was used for tests of statistical significance.

## Results

### Development of the questionnaire

Barriers found by the literature search in the Medline database PubMed were mainly data privacy, poor telematic infrastructure, availability and costs. Facilitators were saving of time, increase of efficiency and an opportunity to bridge distances especially in rural areas. After the pilot test one question was cut out due to lacking anonymity, one question was cut out due to lacking understandability and one subquestion was added due to relevance of a certain scenario (use of telemedicine for tourists, with their GP).

#### Description of the study sample

Of the 2199 questionnaires 699 (response rate: 31.7%) were returned, of which 605 (27.5%) were included in the analysis, 94 of the participants returned the short version of the questionnaire that was included for non-responder analysis instead. Of the participants 507 (84%) were male and 91 (15%) female. A majority of 280 (46%) had a term of office for less than 8 years. Of the communities 462 (77%) were located in a rural area as it is shown in Table. 1. The mayors were also asked if they think their community is located in a rural area, to which agreed 548 (91%).

**Table 1:** Description of the study sample (*n* = 605)

Characteristics	N (%)
**Gender**	
Male	507 (83,8%)
Female	91 (15%)
**Length of term of office**	
< 8 years	280 (46.3%)
8–16 years	177 (29.3%)
17–24 years	70 (11.6%)
Over 24 years	71 (11.7%)
**Number of inhabitants**	
< 1000	249 (41.2%)
1000–2000	87 (14.4%)
> 2000–5000	126 (20.8%)
> 5000–10.000	79 (13.1%)
> 10.000–30.000	52 (8.6%)
> 30.000	10 (1.7%)

#### Telemedicine

The majority of 357 (59%) had a positive/strongly positive opinion about telemedicine. Neutral were 191 (32%) of the participants and negative or strongly negative was chosen by 45 (7%).

The patient communicating with a medical assistant and the GP was supported by 404 (68%) mayors. The telecommunication between the patient with his own telemedicine-device and a doctor (D2P) was ranked appropriate by 388 (64%) participants.

The questionnaire described also a scenario where the patient and his GP communicate with a specialist (D2D), which got 392 (66.8%) positive votes. The fourth scenario described a situation, where patient could bridge the waiting time for an appointment with telemedicine. A positive answer was given by 230 (38.1%) of the participants, 199 (32,9%) were neutral and 167 (27,6%) disagreed. In the last scenario, telemedicine was meant to relieve the local GP during high season, so tourists could consult their own GP - 313 (51.8%) gave that scenario a positive vote, 164 (27.1%) were neutral and 116 (19.1%) had a negative/strongly negative opinion. The aspect of “data privacy” was also requested in the questionnaire, where the mayors were asked what they think about the relevance of data privacy in certain circumstances. In a planned appointment 449 (74,3%) thought it was relevant, 63 (10,4%) thought it wasn’t. In an emergency situation 291 (48,1%) didn’t agree that data privacy was relevant, 112 (15,5%) were neutral and 200 (33%) consider data privacy as relevant.

To these different aspects of telemedicine, the principal component analysis revealed a four-component solution with a total variance (R^2^) of 59.66% (KMO = 0.68, Bartlett’s test for sphericity *P* < 0.001). The Cronbach’s α value for internal consistency was 0.60.

The participants were also asked, whether their community would benefit from telemedicine or not. A majority of 448 (74%) mayors answered, that their community would benefit, 62 (10.2%) mayors disagreed and 83 (13,7%) were neutral. In another question segment, the mayors were asked specifically which group of patients would benefit from telemedicine. Especially the use of telemedicine for follow-up prescription was widely accepted by 543 (89,8%) mayors. As well as the groups of chronically ill patients and bedridden patients, 505 (83.5%) stated, that these two groups would benefit from telemedicine. For patients with upper respiratory infections 461 (76.2%) stated that these patients would benefit and 397 (65.6%) stated that patients in childbed-care would benefit from telemedicine. It was also asked if the mayors think if acute sick patients would benefit from telemedicine, which was denied by 371 (61,3%).

The principal component analysis revealed a two-component solution with a total variance (R^2^) of 40.91% (KMO = 0.78, Bartlett’s test for sphericity *P* < 0.001). The Cronbach’s α value for internal consistency was 0.68.

**Fig. 1:**
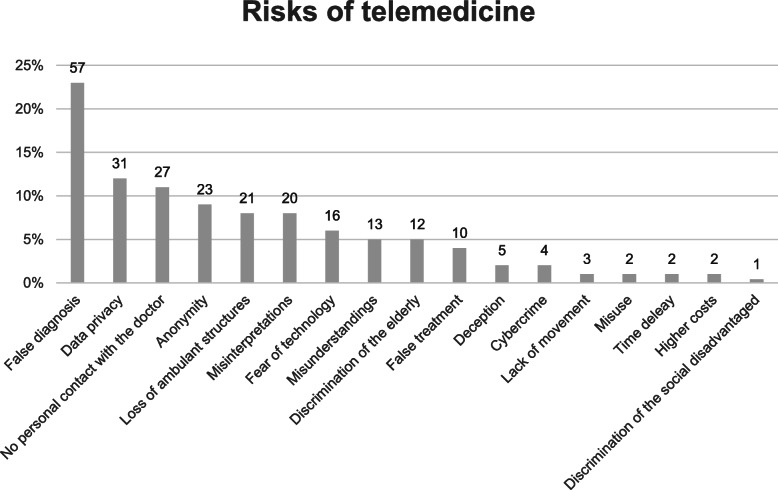
Risks of telemedicine (*n* = 249)

The questionnaire asked if the mayors see risks in treatment with telemedicine as well, which was answered with “yes” by 239 (39,5%) of the participants. A minority of 106 (17,5%) was neutral and 242 (40%) didn’t see risks in telemedicine. The participants who saw risks in telemedicine were asked to describe which kind of risks, which are shown in Fig. 1.

A small majority with about 270 (44,7%) had a lot of trust in telemedicine, 185 (30,6%) were neutral and 86 (14,2%) had little trust in telemedicine.

**Fig. 2:**
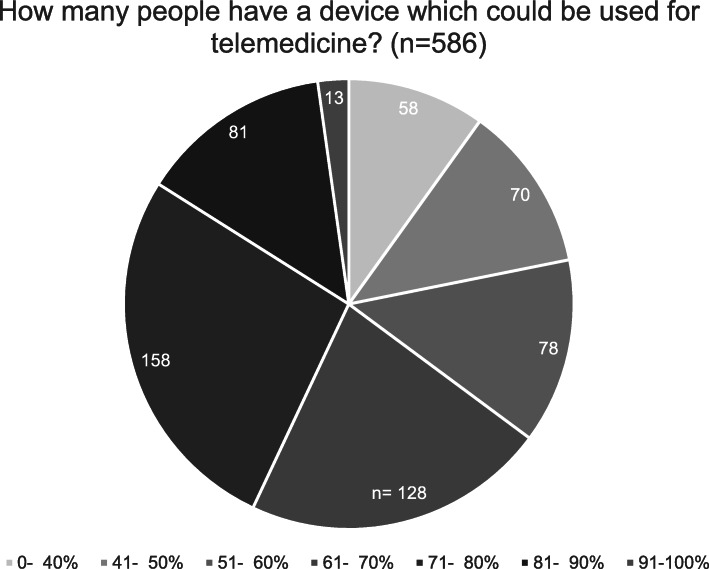
Distribution of telemedicine devices (*n* = 586)

The mayors were also questioned if their community has an internet connection of at least 550 kbit/s. 449 (74,2%) agreed and 121 (20%) disagreed, 28 (4,6%) didn’t know. In order to determine if inhabitants could also use telemedical application without the barrier of purchasing a device, we asked if the mayors could guess how many percent of the inhabitants of the respective community have a device which allows a telemedical treatment, which is also pictured in Fig. 2.

#### General practitioner shortage

**Fig. 3:**
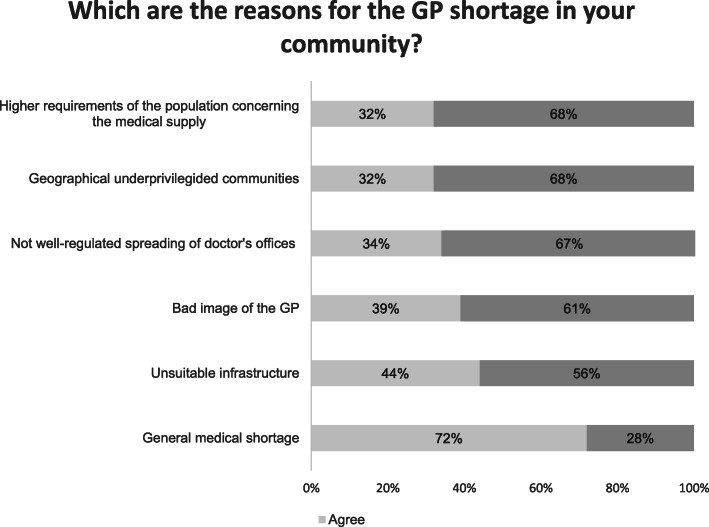
Reasons for GP shortage (*n* = 325)

The questionnaire also included questions about the perceived doctor’s shortage in general and GP shortage in detail. 340 (56,2%) of the mayors thought that there’s no GP shortage in their community, whereas 234 (38,7%) answered that a GP shortage exists. Reasons for the shortage were: A small community, followed by small profits and bureaucracy. Other presumed reasons were high workload for settled GPs, small budgets and geographical reasons like the distance to the next hospital or doctor. For more details, please see Fig. 3.

The mayors were also asked how they would like to prevent GP shortage in their community.

**Fig. 4:**
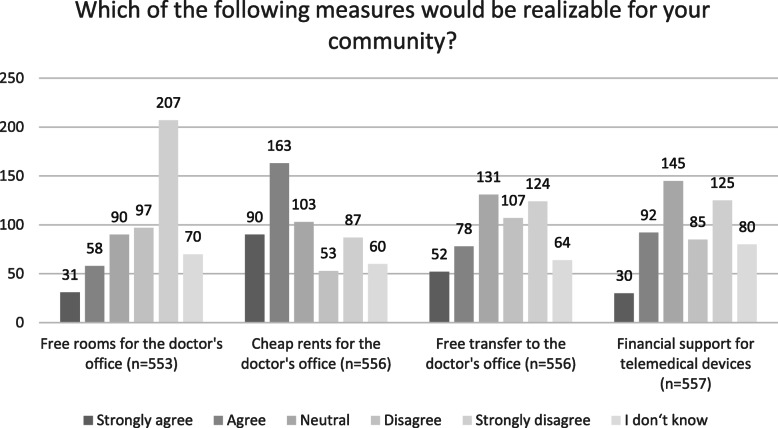
Strategies against GP shortage

On a Likert-scale, they could agree/disagree with four different statements. Figure 4 shows the results in detail.

The questionnaire asked as well how mayors would like to engage in the medical supply for their community. Therefore, different options were presented, 502 (83%) of the participants wished to know about the retirement of the GPs at an early stage. “Early” was for about 50% (*n* = 302) 2–3 years earlier to the retirement date. The initiation of a medical center was supported by 450 (74,4%). Effecting negotiations with the health insurance companies for local issues was wished from around 50% (*n* = 289), 114 (23,8%) were neutral and 155 (25,7%) disagreed. To be involved in the design of telemedical supplies - 179 (29,6%) agreed/strongly agreed, 172 (28,4%) were neutral and 239 (39,5%) disagreed.

The principal component analysis revealed a one-component solution with a total variance (R^2^) of 64.69% (KMO = 0.77, Bartlett’s test for sphericity *P* < 0.001). The Cronbach’s α value for internal consistency was 0.82.

#### Group comparisons

For the group comparisons following results were observed: There were no significant differences (*p* > 0.05) between the gender, mayors from rural areas or cities, and mayors with more years of service than with less concerning perception towards telemedicine.

Mayors from a community with a perceived GP shortage had a more negative perception of telemedicine than communities without this perception (*p* = 0.020).

#### Non-responder analysis

Of the 94 non-responder 6 (6.4%) were female and 88 (93.6%) male, the stated age ranged from 27 years to 81 years and 74 (78.7%) of the non-responder stated their community is in a rural area. The length of term of office ranged from 0.5 years to 40 years. Reasons for the non-participation were following: lack of relevance of that topic (38.3%), rejection of telemedicine (23.4%) no participation in surveys in general (22.3%) and lacking interest in that topic (3.2%).

## Discussion

Aim of this study was to explore the perception of representatives of the local government regarding telemedicine and the perceived GP shortage. The different items of the questionnaire are reflected in the sections as shown by the principal component analysis. Moreover, an acceptable internal consistency for the different sections was observed. The main finding is the generally risen acceptance towards telemedicine compared to former surveys with this target audience as only about 10% had a negative perception about telemedicine. The majority of the participants thought their community would benefit from telemedicine and most participants had a positive opinion about it themselves. These results were particularly impressive because in a study from 2017 only 14% of representatives of the local government of the federal state of Lower Saxony in Germany had a positive opinion about telemedicine [18].

Another difference between these two studies was the aspect of data security, which was compared to our data a minor issue in 2017 [18]. Data security is a subject which is frequently discussed. Especially compared to other countries, which are way more advanced when it comes to telemedicine [28]. It is a quite emotional topic, especially because health data is considered most sensitive [29].

Remarkable finding for future studies is the fact, that in our data communities from perceived rural areas had a more negative perception towards telemedicine. This is in an important issue for decision-makers when it comes to providing healthcare via telemedicine in the future.

Interestingly depending on the degree of urgency, our participants judged data security as less important. In addition to the European General Data Protection Regulation (GDPR) [30] there are national data protection regulations. These juristic guidelines unfold a complex condition, especially for IT providers that address the professional secrecy for health professions [29, 31]. Therefore, the National Association of Statutory Health Insurance certifies selected telemedicine providers. At the moment there are 27 certified providers according to the official website of the National Association of SHI [32].

Another common concern and therefore barrier named was the fear of anonymity, which might lead to a worse doctor-patient relationship. There is evidence that continuity of care is indeed a crucial part of primary care. Studies show that higher continuity of care leads to a greater patient satisfaction, increased compliance and improvement of the mortality rates [33, 34].

Furthermore, there were concerns that telemedical treatment might be inferior to conventional practice. The mayors expressed the fear of false diagnoses. Also, medical professionals and medical students were skeptical about telemedical applications for disease monitoring [31]. Evidence regarding the quality of telemedicine care is heterogeneous. E.g. a former study compared patients with diabetic foot ulcers, which were either monitored via telemedicine or treated with a standard outpatient monitoring. The group of patients who were telemonitored showed higher mortality [35]. Whereas a review addressing the treatment of heart failure had similar outcomes depending on whether telemedicine or the face-to-face communication was used [36].

### Perspective

The year 2020 took many unexpected turns, which weren’t predictable and had a huge impact all around the world. The Covid-19 pandemic changed the life of many and also changed the usage of telemedicine. When our questionnaire was developed, this certain situation wasn’t considered, but as the developments show, telemedicine became one important tool.

Due to the recent SARS-CoV-2 pandemic, telemedicine became a treatment of particular importance In New York, the use of telemedicine increased from around 500 a day to more than 8500 telemedical visits a day within a span of 1 month [37]. The SARS-CoV-2 is a virus which is transmitted over aerosols and droplet infections [38], therefore one of the main keys in preventing infection is the reduction of contacts. With telemedicine, patients could be treated from home and therefore reduce their out of house movements. But not only patients are helped by this, it also protects medical staff. And with fewer patients in the doctor’s offices, another advantage is the saving of protective clothing, masks and disinfectants, goods which are short in this crisis [39]. This crisis shows that telemedicine holds much potential, but it’s also a very special situation [40].

In that prospect our findings towards the willingness to use telemedicine in case of an emergency was high fits to these developments during the pandemic.

Also due to the pandemic with its higher mortality within physicians, GP shortage may be even more severe in the future [41]. In addition to this actual or upcoming GP shortage, telemedicine seems to offer an addition to previous known circumstances. However, our finding, that especially those communities with a perceived shortage do accept telemedicine the least needs to be taken into account for future projects.

### Strengths and limitations

This is one of the first studies, which inquires the opinion of local politicians towards telemedicine in a broad extent, as in former studies with mayors the focus wasn’t on telemedicine. But due to the political influences of the mayors in connection to the medical supply in each community, this study wanted to explore their specific opinions. The response rate was lower compared to the survey in Lower Saxony in Germany where mayors were queried in the past [16–18]. Due to this high non-response selection-bias can’t be ruled out. Results can’t be generalized due to their descriptive nature. The participants of this study were representatives of the local governments in Schleswig-Holstein and Baden-Württemberg and not the citizens or medical professionals. Due to constraints of the ethical commission, more detailed information about the participants including their county and age was restricted. Therefore, comparisons between the subgroups weren’t possible. As the number of male mayors in both federal states are 83.8% the number of male participants was relatively higher than the females. Therefore a gender comparison would have not been meaningful. Even though we included two regional extreme regions it was a cross-sectional study, and thus, we must be cautious to derive causal links from these findings.

## Conclusion

The acceptance of telemedicine is rising. In this study, known barriers like data security concerns were seen as less important in case of an emergency, the highest risk of telemedicine was seen in misdiagnosing. However, our results of the group comparisons show that telemedicine is not seen as an alternative for communities with an actual perceived GP-shortage.

## Supplementary Information

**Additional file 1.** : Questionnaire, English version.

## Data Availability

The datasets used and/or analyzed during the current study are available from the corresponding author on reasonable request.

## Abbreviations

BBSR: The Federal Office of Construction and Urban Research
D2D: Doctor-to-Doctor
D2P: Doctor-to-Patient
GDPR: European General Data Protection Regulation
MAST: Model for Assessment of Telemedicine
IT: Information Technology
SHI: Statutory Health Insurance
