# Supplementary material for: Facilitators and barriers for the implementation of telemedicine from a local government point of view - a cross-sectional survey in Germany
Source: BMC Health Serv Res. 2021 Sep 6;21:919. doi: 10.1186/s12913-021-06929-9 (PMC8419374; doi:10.1186/s12913-021-06929-9)
Supplement: Supplementary file 1 — Additional file 1. : Questionnaire, English version. [file 12913_2021_6929_MOESM1_ESM.docx]

**View of the municipality on telemedicine* applications and future healthcare close to home.**

Dear Mayor,

The future of healthcare access, especially close to home, is a central issue. Telemedical applications could support care close to home. For these services, legal, ethical, and organisational aspects should be considered in advance. With this survey, we would, therefore, like to find out your perception/opinion towards different aspects of residential care and telemedicine.

* In the following, telemedicine is a collective term for various medical care concepts, which have in common the principle approach that provides the medical services of the health care of the population in the areas of diagnostics, therapy and rehabilitation as well as in medical decision guidance over spatial distances (or time shifts). Information and communication technologies are used for this purpose.

(Definition by the AG Telemedizin der Bundesärztekammer)

The survey is anonymous. By completing the questionnaire, you agree that your answers will be scientifically evaluated by the Institute of General Medicine of the University Medical Center Schleswig-Holstein at the Campus Lübeck. Your information will be treated in strict confidence and the data will not be passed on to third parties. The questionnaire contains 20 questions which collectively takes approximately 20 minutes to answer. The results of this survey will be used to further optimize healthcare access close to home.

Please note the following information when filling out the form:

• Please use only blue or black ballpoint pens.

• Please enter free text in block letters.

• Please tick the boxes clearly: ⌧

• In case of accidental wrong selection, please fill in the corresponding box completely: ◼

and tick your selection again: ⌧

If you have any questions regarding this survey, please contact us:

Prof. Dr. med. Jost Steinhäuser

Tel: 0451 3101 8001

Email: jost.steinhaeuser@uksh.de

1. **Perception of telemedicine.**

| **Strongly positive** | | | | | **Strongly negative** | | |
| --- | --- | --- | --- | --- | --- | --- | --- |
|  | **1** | **2** | **3** | **4** | | **5** |  |
| What is your perception of telemedicine in general? | □ | □ | □ | □ | | □ |  |

1. **How suitable do you consider the following telemedicine scenarios to be for enabling as many patients as possible to access telemedical services?**

| **Very suitable** | | | | | **Not suitable at all** | | |
| --- | --- | --- | --- | --- | --- | --- | --- |
|  | **1** | **2** | **3** | **4** | | **5** |  |
| **a.** Patient communicates with his own telemedical device with the doctor. | □ | □ | □ | □ | | □ |  |
| **b.** Patient communicates with a medical assistant, who provides the telemedical device, and with the doctor. | □ | □ | □ | □ | | □ |  |
| **c.** Patient and the GP communicate via the device, provided by the GP, with a specialist. | □ | □ | □ | □ | | □ |  |

| **d.** Patient bridges the waiting time for an appointment with a specialist (e.g. psychotherapist) with telemedical aids. | □ | □ | □ | □ | □ |  |
| --- | --- | --- | --- | --- | --- | --- |
| **e.** Telemedicine could be used to relieve the doctors at a holiday resort by allowing tourists to consult their family doctor via telemedicine. | □ | □ | □ | □ | □ |  |

1. **Basic principles.**

|  | | Yes | No | | I don’t know | |
| --- | --- | --- | --- | --- | --- | --- |
| **a.** Does your municipality have an Internet connection of at least 550 kbit/s? | □ | | □ | □ |  |  |
| If so, what is the percentage of the population in your municipality that  has access to this Internet connection? | **** | | % | □ |  |  |
| **b.** Do you think your community would benefit from telemedicine? | □ | | □ | □ |  |  |
| **c.** Are there homes in your community that are equipped with Ambient Assisted Living* devices? | □ | | □ | □ |  |  |
| **d.** Do you consider telemedicine to be a relevant economic sector in Germany? | □ | | □ | □ |  |  |
| **e.** Do you see telemedicine as a new type of care concept? | □ | | □ | □ |  |  |
| **f.** Do you see risks for your citizens in telemedicine?  If so, which ones________________________________________________________ | □ | | □ | □ |  |  |
| **g.** Should your citizens be actively motivated to participate in telemedical projects? | □ | | □ | □ |  |  |

***** Ambient Assisted Living stands for age-appropriate assistance systems for a healthy and independent life. An example of this is a sensor that measures that a patient has fallen in his or her home and thus automatically activates assistance (e.g. the neighbor).

1. **Do the following patient groups benefit from telemedicine in your opinion?**

|  | | Yes | No | | I don’t know | |
| --- | --- | --- | --- | --- | --- | --- |
| **a.** Acute sick (e.g. patients with a stroke) | □ | | □ | □ |  |  |
| **b.** Chronic sick (e.g. patients with a type II diabetes) | □ | | □ | □ |  |  |
| **c.** Mobility impaired patients (e.g. bedridden patients) | □ | | □ | □ |  |  |
| **d.** Patients with chronic wounds | □ | | □ | □ |  |  |
| **e.** Patients for aftercare post an operation (e.g. after eye surgery) | □ | | □ | □ |  |  |
| **e.** Support for families in the postpartum period | □ | | □ | □ |  |  |
| **f.** Patients with common infections of the upper respiratory tract | □ | | □ | □ |  |  |
| **g**. Patient with follow-up prescription request | □ | | □ | □ |  |  |
| **h.** Certificate of incapacity for work | □ | | □ | □ |  |  |

1. **Please rate the following statements.**

| **Very motivated** | | | | | **Not at all motivated** | | |
| --- | --- | --- | --- | --- | --- | --- | --- |
|  | **1** | **2** | **3** | **4** | | **5** |  |
| **a.** How motivated do you think your citizens are to receive telemedical treatment in addition to the current medical services? | □ | □ | □ | □ | | □ |  |
| **b.** How motivated are you to undergo telemedical treatment yourself? | □ | □ | □ | □ | | □ |  |

1. **What is your estimate: What percentage of your citizens have a device (e.g. a smartphone, tablet) with which they can directly participate in a telemedical offer?**

**_______________ %**

1. **How much money do you think a doctor should receive for a telemedicine consultation?**

| **More* Same*** | | | | | **Less*** | | |
| --- | --- | --- | --- | --- | --- | --- | --- |
| □ **_________________________**□ |  |  |  |  | | □ |  |

*than a regular consultation

1. **Rate the following statement.**

| **Great** | | | | | **None** | | |
| --- | --- | --- | --- | --- | --- | --- | --- |
|  | **1** | **2** | **3** | **4** | | **5** | I don’t know |
| How would you describe your trust in telemedically supported medical care? | □ | □ | □ | □ | | □ | □ |

1. **How relevant do you consider the issue of data protection in the following situations?**

| **Very relevant** | | | | | **Not relevant at all** | | |
| --- | --- | --- | --- | --- | --- | --- | --- |
|  | **1** | **2** | **3** | **4** | | **5** |  |
| **a.** A planned consultation | □ | □ | □ | □ | | □ |  |
| **b.** An emergency | □ | □ | □ | □ | | □ |  |

1. **How much would you like to become active for your community on the following topics?**

| **A lot** | | | | | **Not at all** | | |
| --- | --- | --- | --- | --- | --- | --- | --- |
|  | **1** | **2** | **3** | **4** | | **5** |  |
| 1. Learning early on that a doctor is looking for a successor | □ | □ | □ | □ | | □ |  |
| What would you consider to be "early"? _____Years |  |  |  |  | |  |  |
| 1. When initiating a communal ambulatory health care center | □ | □ | □ | □ | | □ |  |
| 1. To be able to negotiate directly with health insurers for aspects of local care | □ | □ | □ | □ | | □ |  |
| 1. To be more involved in the design of telemedical services | □ | □ | □ | □ | | □ |  |

1. **Please estimate…**

|  | | Yes | No | | I don’t know | |
| --- | --- | --- | --- | --- | --- | --- |
| **a.** Is your community located in a rural area? | □ | | □ | □ |  |  |
| **b.** Is there a lack of medical specialists (e.g. ophthalmologists) in your community? | □ | | □ | □ |  |  |
| **c.** Is there a GP shortage in your community? | □ | | □ | □ |  |  |

1. **If there is a GP shortage in your community, what do you think are the reasons for this?**

*Multiple answers possible*

|  | |  |  | | |  | |
| --- | --- | --- | --- | --- | --- | --- | --- |
| **a.** Higher requirements of the population concerning the nearby medical supply |  | | □ |  | |  |  |
| **b.** General GP shortage |  | | □ |  | |  |  |
| **c.** Bad image of the GP profession |  | | □ |  | |  |  |
| **d.** The infrastructure of the municipality is unattractive for the demands of the upcoming generation of doctors |  | | □ |  | |  |  |
| **e.** The municipality is geographically underprivileged |  | | □ |  | |  |  |
| **e.** Not well-regulated spreading of doctor’s offices |  | | □ |  | |  |  |
| **f.** Other reasons _____________________________________________________ |  | | □ |  | |  |  |

1. **What measures could be implemented in your community to attract a general practitioner to settle?**

| **Very likely** | | | | | **Not likely at all** | | |
| --- | --- | --- | --- | --- | --- | --- | --- |
|  | **1** | **2** | **3** | **4** | | **5** | I don’t know |
| **a.** Free rooms for the doctor’s office | □ | □ | □ | □ | | □ | □ |
| **b.** Cheap rents for the doctor’s office | □ | □ | □ | □ | | □ | □ |
| **c.** Free transfer to the doctor’s office | □ | □ | □ | □ | | □ | □ |
| **d.** Financial support for telemedical devices | □ | □ | □ | □ | | □ | □ |

1. **Assessment of journey times.**

| The travel time is from your town hall by car... | <10 min | 11- 30 min | 31- 60 min | >60 min |
| --- | --- | --- | --- | --- |
| **a.** to the next GP | □ | □ | □ | □ |
| **b.** to the closest hospital | □ | □ | □ | □ |
| **c.** to the closest pharmacy | □ | □ | □ | □ |
| **d.** to the closest supermarket | □ | □ | □ | □ |
| **e.** to the closest school | □ | □ | □ | □ |
| **f.** to the closest kindergarten | □ | □ | □ | □ |
| **g.** to the closest movie theater | □ | □ | □ | □ |

1. **You are…** □ male □ female
2. **How many years have you been mayor?** □< 8 □ 8-16 □ 17-24 □ > 24 Jahre
3. **How often have you been a patient to a doctor in the last 12 months? ____________**
4. **How many inhabitants does your municipality have?**

□< 1000 □ 1000-2000  >2000-5000 □ > 5000-10.000 □>10.000-30.000 □>30.000

1. **Was there one important aspect missing from this survey? If so, which one?**

___________________________________________________________________________________

___________________________________________________________________________________

___________________________________________________________________________________

___________________________________________________________________________________

___________________________________________________________________________________

**Many thanks for your participation!**

**Information on non-participation in the study**

If you do not wish to take part in this survey, you would be very helpful to us if you would provide the following information on non-participation:

| Gender: | □ female □ male |
| --- | --- |
| Age: |  |
| Is your community located in a rural area? | Yes □ No □ |
| How many years have you been mayor? |  Years |
| Reasons for non-participation: (multiple entries are possible) |  The subject does not interest me   The subject is not relevant for me   I am generally opposed to telemedicine   I generally do not participate in surveys   others ………………………….……. |

Thanks a lot!
